# Piezo acts as a molecular brake on wound closure to ensure effective inflammation and maintenance of epithelial integrity

**DOI:** 10.1016/j.cub.2022.06.041

**Published:** 2022-08-22

**Authors:** Luigi Zechini, Clelia Amato, Alessandro Scopelliti, Will Wood

**Affiliations:** 1 Centre for Inflammation Research, Queens Medical Research Institute, University of Edinburgh, 47 Little France Crescent, Edinburgh EH16 4TJ, UK

**Keywords:** wound healing, Piezo, inflammation, epithelial resilience, calcium, ROS, Drosophila

## Abstract

Wound healing entails a fine balance between re-epithelialization and inflammation^1^^,^^2^ so that the risk of infection is minimized, tissue architecture is restored without scarring, and the epithelium regains its ability to withstand mechanical forces. How the two events are orchestrated *in vivo* remains poorly understood, largely due to the experimental challenges of simultaneously addressing mechanical and molecular aspects of the damage response. Here, exploiting *Drosophila*’s genetic tractability and live imaging potential, we uncover a dual role for Piezo—a mechanosensitive channel involved in calcium influx^3^—during re-epithelialization and inflammation following injury *in vivo*. We show that loss of Piezo leads to faster wound closure due to increased wound edge intercalation and exacerbated myosin cable heterogeneity. Moreover, we show that loss of Piezo leads to impaired inflammation due to lower epidermal calcium levels and, subsequently, insufficient damage-induced ROS production. Despite initially appearing beneficial, loss of Piezo is severely detrimental to the long-term effectiveness of repair. In fact, wounds inflicted on *Piezo* knockout embryos become a permanent point of weakness within the epithelium, leading to impaired barrier function and reduced ability of wounded embryos to survive. In summary, our study uncovers a role for Piezo in regulating epithelial cell dynamics and immune cell responsiveness during damage repair *in vivo*. We propose a model whereby Piezo acts as molecular brake during wound healing, slowing down closure to ensure activation of sustained inflammation and re-establishment of a fully functional epithelial barrier.

## Highlights

• Loss of Piezo accelerates wound closure and weakens inflammation *in vivo*
• Loss of Piezo enhances wound edge cell intercalation and myosin cable heterogeneity
• Loss of Piezo reduces epidermal Ca^++^ levels and damage-induced ROS production
• Loss of Piezo compromises post-wounding epithelial barrier function and survival

## Graphical abstract

**Figure:**
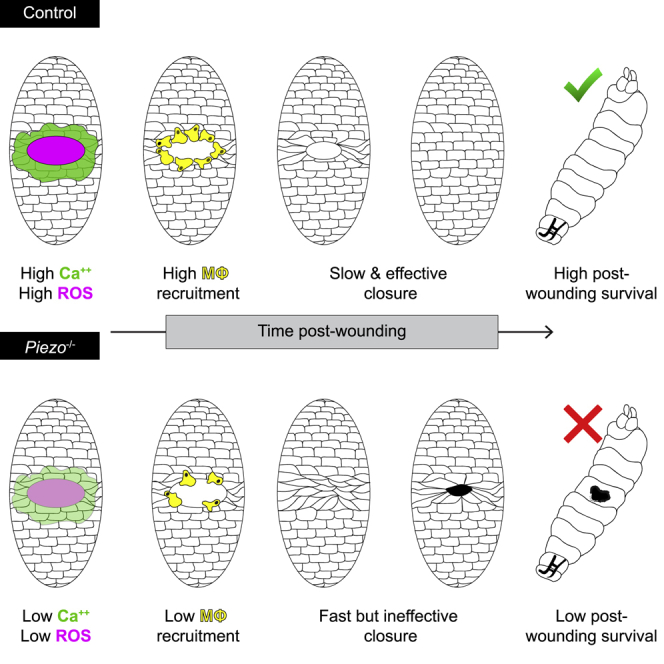

## Teaser

Zechini et al. have shown that the mechanosensitive channel Piezo delays wound closure by limiting myosin cable heterogeneity and wound edge cell intercalation, while also ensuring a sustained inflammatory response. Piezo is essential for efficient restoration of epithelial barrier function and to ensure post-wound survival *in vivo*.

## Results and discussion

### Loss of Piezo accelerates epithelial wound closure

**Figure 1:**
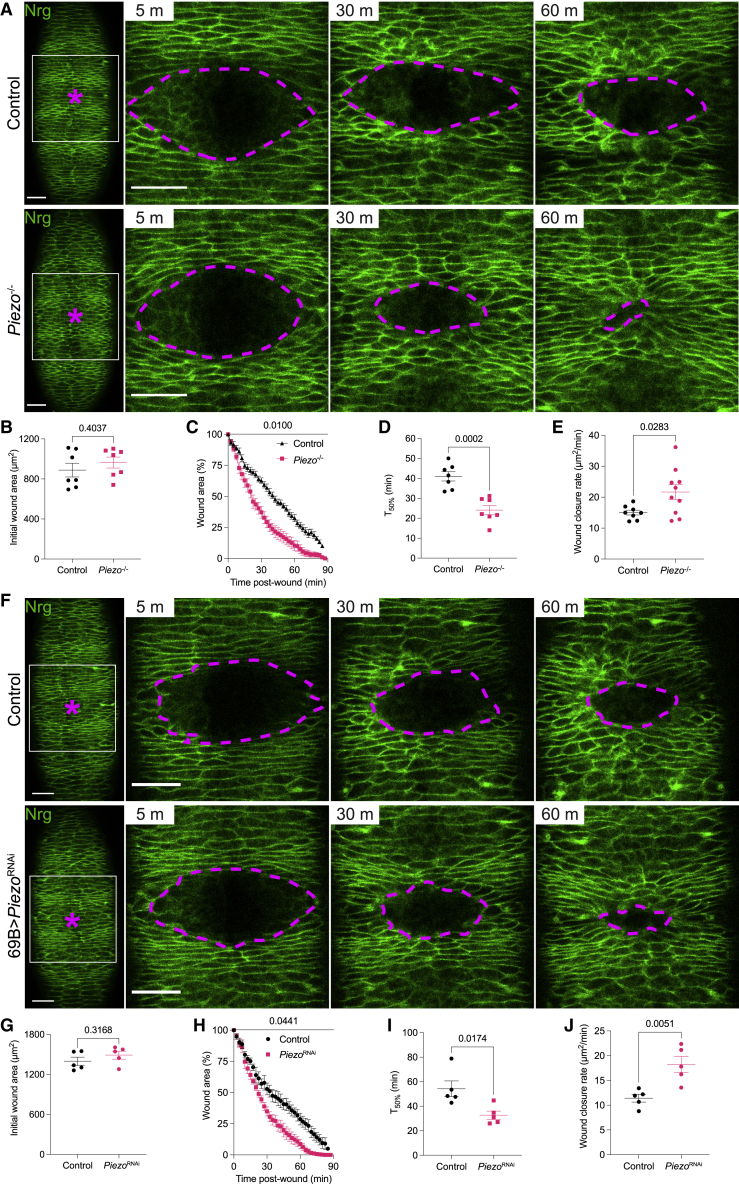
Loss of Piezo accelerates epithelial wound closure (A and F) Confocal time-lapse of stage 15 embryos expressing Nrg to mark epidermis (green). Asterisk: wound. Time-points refer to minutes post-wounding. Dashed line: wound outline. (B and G) Initial wound area. (C and H) Closure progression from wounds’ maximal extension. (D and I) Time required for wounds to reach 50% of maximal size. (E and J) Rate of wound closure. Scale bars, 20 μm; error bars: SEM. Related to Figure S1. See Videos S1 and S2. Video S1. Loss of Piezo accelerates epithelial wound closure, related to Figure 1A*Piezo*^-/-^ embryos close epithelial wounds faster than control embryos. Epithelium (green): Nrg; asterisk: wound; scale bars: 20 μm Video S2. Loss of Piezo within the epithelium accelerates wound closure, related to Figure 1FEpithelial *Piezo*^RNAi^ embryos close epithelial wounds faster than control embryos. Epithelium (green): Nrg; asterisk: wound; scale bar: 20 μm

To create reproducible wounds on the ventral epithelium of *Drosophila* embryos, we used a well-established laser ablation assay^2^ followed by confocal live imaging to monitor wound closure. Analysis of wounds inflicted to stage 15 control and *Piezo* knockout (*Piezo*^-/-^) embryos labeled with the GFP protein trap for the septate junction component neuroglian (Nrg) revealed that loss of Piezo strikingly accelerates wound closure (Figure 1A). Measuring wound size throughout repair clearly showed that, despite a comparable initial area (Figure 1B), wounds generated in *Piezo*^-/-^ embryos close significantly faster than those in control embryos (Figure 1C). Accordingly, a reduced T_50%_ (time required to reach 50% of the maximal size) and an increased wound closure rate (area of wound closed over time) is observed in *Piezo*^-/-^ when compared to control embryos (Figures 1D and 1E). We then asked within which tissue Piezo exerts its wound closure-regulating function. Since Piezo is involved in sensing changes in mechanical forces,^4^^,^^5^ and wounding represents a dramatic mechanical input, we hypothesized that Piezo regulates wound closure by acting within the epidermis. To understand whether Piezo is expressed within this tissue, we performed immunostaining of embryos carrying a MiMIC-based GFP protein trap^6^ construct within the *Piezo* locus, which revealed Piezo’s prominent expression at the plasma membrane of ventral epidermal cells (Figure S1A). Using the GAL4/UAS system,^7^ we then specifically suppressed *Piezo* expression within the epidermis (*Piezo*^RNAi^), which resulted in a significant reduction in Piezo expression (Figures S1B and S1C) and a comparable acceleration of wound closure (Figure 1F). Again, despite comparable initial areas (Figure 1G), wounds made to *Piezo*^RNAi^ embryos showed reduced T_50%_ and faster wound closure rate (Figures 1I and 1J). Taken together, these data demonstrate that Piezo is expressed in the *Drosophila* embryonic epidermis and that its loss within this tissue accelerates wound closure, suggesting a model whereby epidermal Piezo acts cell-autonomously as a molecular brake to slow down re-epithelialization. Lastly, we asked whether loss of Piezo impacts other aspects of epithelial homeostasis and dynamics, including maintenance of cell numbers across the embryonic epidermis and effectiveness of dorsal closure—a morphogenetic episode that has been shown to parallel several aspects of wound repair.^2^^,^^8^ As shown in Figures S1D–S1G, we found that loss of Piezo does not lead to any significant difference in epidermal cell number, nor to an impairment of dorsal closure.

### Loss of Piezo locally increases tissue fluidity and exacerbates myosin cable heterogeneity

**Figure 2:**
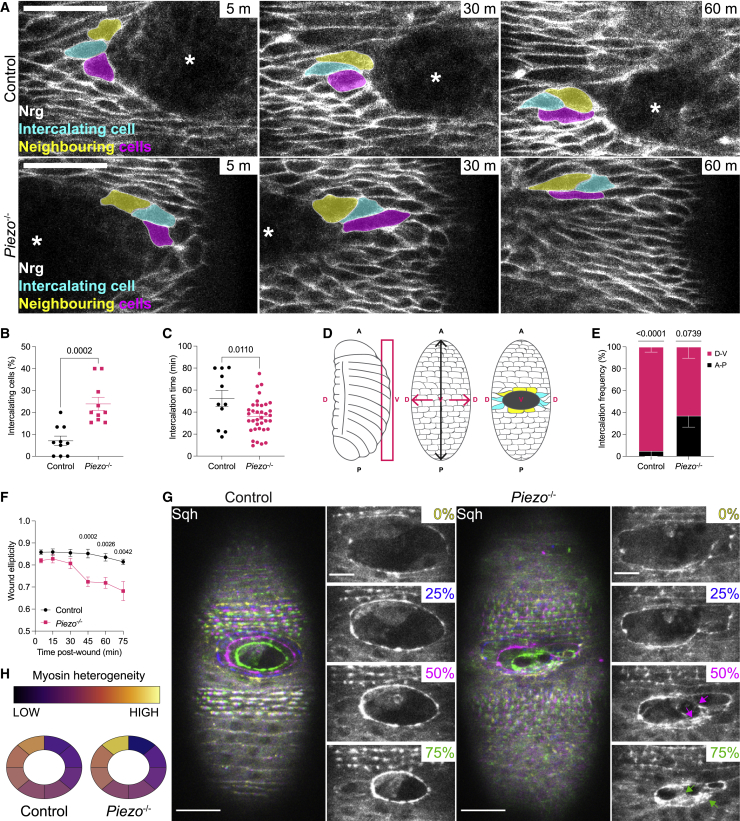
Loss of Piezo increases tissue fluidity and exacerbates myosin heterogeneity at the wound edge (A) Examples of wound edge intercalation. Nrg (gray): epithelium; cyan: intercalating cells; yellow/magenta: neighboring cells. (B) Percentage of intercalating cells. (C) Time required for intercalation. (D) Schematic representation of a stage 15 embryo with A-P/D-V axes indicated. Left: lateral view, ventral side highlighted by magenta box. Middle/right: ventral view of an unwounded/wounded (black ellipse) embryo. Relative position of epidermal cells facing the wound: cyan: cells along the D-V axis; yellow: cells along the A-P axis. (E) Percentage of intercalation events along the A-P/D-V axes. (F) Wound ellipticity during closure. (G) Confocal images of wounded embryos expressing Sqh-GFP (gray) to outline the wound edge. Set time-points, corresponding to percentage of wound closure, are shown in the insets and superimposed as pseudo-color on the maximal extension to highlight closure progression. (H) Heatmap of normalized myosin intensity along the wound edge at 50% of closure to visualize myosin heterogeneity. Scale bars, (A) (G): 20 μm; (G, inset): 10 μm. Error bars: SEM. Related to Figure S2.

Which epithelial properties, affected by the loss of Piezo, could account for faster re-epithelialization? Like different morphogenetic processes, wound healing requires a certain degree of epithelial mechanical adaptation.^2^^,^^9^ Specifically, for wounds to close effectively, epithelial cells in direct contact with the damaged area need to be able to slide past their neighbors—progressively withdrawing from the wound edge—in a process termed “wound edge intercalation.”^10^ Therefore, we asked whether loss of Piezo increases epithelial cells’ ability to intercalate. Tracking of individual wound edge-facing epithelial cells revealed that, despite occurring in both genotypes (Figure 2A), intercalation is more frequent and faster in *Piezo*^-/-^ embryos (Figures 2B and 2C). We also asked whether loss of Piezo affects the spatial distribution of intercalation events. Epidermal cells display an anisotropic morphology, with an extended margin along the embryonic dorso-ventral (D-V) axis, and a narrow margin along the embryonic antero-posterior (A-P) axis. Cells located along the A-P axis face the wound by their longest sides, while those along the D-V axis face it by their shortest sides (Figure 2D). In order to intercalate, the former need to re-establish cell-cell contacts on a longer stretch of plasma membrane compared to the latter, which should render intercalation along the A-P axis a far greater challenge. Accordingly, in control embryos, the vast majority of wound-edge intercalation events occur along the D-V axis. However, in *Piezo*^-/-^ embryos, a larger proportion of intercalation events occur along the embryonic A-P axis—and independently of their relative position to the wound (Figure 2E). These results indicate that loss of Piezo facilitates the transition from a solid-like (jammed) to a more fluid-like (unjammed) epithelium upon wounding. We wondered whether such fluidization affects the entire epithelium or is instead localized to the wound-surrounding epithelial cells. To address this question, we tracked individual epithelial cells two rows away from the damaged area and found no increase in the rate of intercalation (Figure S2A)—suggesting that loss of Piezo triggers a regionalized, rather than a tissue-wide, increase in fluidity. Further supporting this notion, we found no difference in overall epithelial tension—as measured by cortical myosin levels—in unchallenged epithelia (Figure S2B). Wound-facing epithelial cells respond quickly to tissue damage by mobilizing their cytoskeletal components to assemble an actomyosin cable, a supracellular structure that provides the force to coordinate cell contraction—ensuring a seamless closure^1^—while also conferring an elliptic shape to the healing wound. While control wounds remain elliptic as they close, *Piezo*^-/-^ wounds gradually lose symmetry (Figure 2F). We asked whether such irregularity could be linked to a dysfunctional actomyosin cable assembly. To address this, we monitored myosin dynamics at the wound edge during re-epithelialization, which showed that a myosin-rich cable is swiftly assembled around the wound in both genotypes (Figure 2G) with no measurable difference in myosin enrichment (Figures S2C and S2D). However, while myosin remains confined within the wound outline in control embryos, its distribution along the wound in *Piezo*^-/-^ embryos appeared more disorganized, with myosin clusters (arrows) forming as the wound closes. Intriguingly, myosin heterogeneity has been previously reported to facilitate wound closure,^9^^,^^11^ which prompted us to ask whether loss of Piezo exacerbates this heterogeneity, contributing to a faster re-epithelialization. To quantitate myosin heterogeneity around the wound edge, we measured its intensity along eight equally long segments and normalized the averaged intensity values. Strikingly, we found that myosin heterogeneity around the wound perimeter is greatly enhanced in *Piezo*^-/-^ embryos (Figures 2H, S2E, and S2G). Altogether, our data show that loss of Piezo accelerates re-epithelialization by locally increasing tissue fluidity around the wound edge—leading to more frequent, faster, and more widespread intercalation events—and by exacerbating myosin cable heterogeneity.

### Loss of Piezo reduces epithelial calcium levels, affects damage-induced ROS production, and weakens the inflammatory response

**Figure 3:**
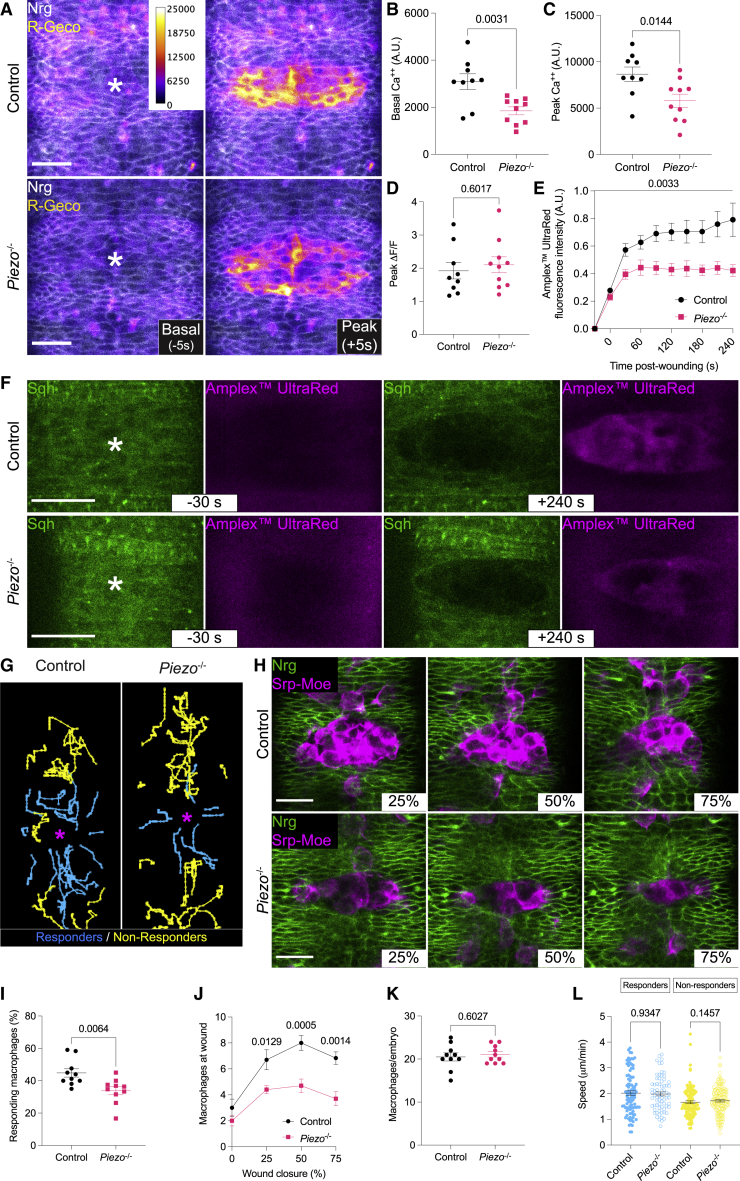
Loss of Piezo reduces the wound-induced inflammatory response (A) Confocal images of basal (pre-wounding) and peak (post-wounding) calcium flux across the ventral epidermis; asterisk: wound. (B) Basal (pre-wounding) calcium levels. (C) Peak (post-wounding) calcium levels. (D) Normalized peak (post-wounding) calcium levels. (E) Normalized Amplex™ UltraRed intensity fluorescence prior to and post-wounding. (F) Confocal images of unwounded (-30 s) and wounded (+240 s) control and *Piezo*^-/-^ embryos expressing an epithelial marker (Sqh, green) and injected with Amplex™ UltraRed (magenta); asterisk: wound. (G) Macrophages tracked for 90 minutes after embryo wounding; asterisk: wound; cyan tracks: responding macrophages, yellow tracks: non-responding macrophages. (H) Confocal images of macrophages (magenta) recruited to the wound site; epithelium: Nrg, green. (I) Percentage of responding macrophages within individual wounded embryos. (J) Recruitment of macrophages to wounds at defined wound closure percentages. (K) Total number of macrophages within individual wounded embryo. (L) Speed of individual responding (cyan) and non-responding (yellow) macrophages in wounded embryos. Scale bars, 20 μm; error bars: SEM. Related to Figure S3. See Videos S3 and S4. Video S3. Loss of Piezo leads to reduced basal calcium levels but does not impair calcium wave propagation, related to Figure 3A*Piezo*^-/-^ embryos have lower basal (pre-wounding) calcium levels than control embryos, but comparable calcium wave propagation upon wounding. Epithelium (gray): Nrg; calcium reporter (fire LUT): R-Geco; asterisk: wound; scale bars: 20 μm Video S4. Loss of Piezo leads to reduced inflammatory response, related to Figure 3H*Piezo*^-/-^ embryos show a reduced number of macrophages recruited to wounds in comparison to control embryos. Epithelium (gray): Nrg; macrophages (green): Srp-Moe-Cherry; scale bars: 20 μm

Piezo is a non-selective cation channel^3^ mainly involved in calcium influx. Since calcium is a conserved early damage signal—spreading as an instantaneous wave across epithelia in response to wounds—we asked whether loss of Piezo affects calcium dynamics and/or levels within epidermal cells. To achieve this, we expressed two independent calcium reporters, R-Geco and GCaMP3, within the epithelium of control and *Piezo*^-/-^ embryos. Visual analysis indicated that Piezo is not required for the generation of a damage-induced calcium wave (Figures 3A and S3A). However, quantitative measurements revealed a dramatic reduction in basal calcium levels across unwounded *Piezo*^-/-^ epithelia (Figures 3B and S2B), which, in turn, resulted in a significant decrease in damage-induced calcium peak levels across wounded epithelia (Figures 3C and S3C). Therefore, while Piezo does not mediate damage-induced calcium influx and spread, it plays a role in maintaining homeostatic epidermal calcium levels. Despite no significant difference in the normalized peak calcium intensity (Figures 3D and S3D), we hypothesized that the overall lower peak calcium levels across the epidermis may hinder the activation of downstream damage responses. A conserved calcium-dependent event that follows tissue damage is the production of ROS within the wounded epithelium, which is mediated by the calcium-responsive NADPH dual oxidase (DUOX)^1^. Therefore, we asked whether loss of Piezo affects the production of damage-induced ROS. Wounding embryos injected with the fluorescent ROS reporter Amplex™ UltraRed revealed a striking reduction of damage-induced ROS production upon loss of Piezo (Figures 3E and 3F). To understand whether this reduction was due to decreased calcium levels, we knocked down plasma membrane calcium-ATPase (PMCA) within the *Piezo*^-/-^ embryonic epidermis, since suppression of this calcium export pump has been shown to restore cytoplasmic calcium levels and revert the effects of *Piezo* loss in the *Drosophila* gut.^12^ As expected, suppressing *PMCA* expression is sufficient to reinstate damage-induced ROS production in *Piezo*^-/-^ embryos (Figures S3E and S3F). ROS such as H_2_O_2_ act as a potent damage signal for immune cells and are essential for their recruitment to sites of tissue damage—where they fulfill the fundamental role of clearing debris and pathogens. We therefore sought to understand whether loss of Piezo impairs immune cell recruitment. Tracking of individual macrophages throughout closure revealed a significant reduction in the number of immune cells recruited to wounds (Figures 3G–3J). Importantly, this effect was not caused by an overall reduction in the number of macrophages within the embryo, nor by an inherent defect in migratory speed—either between responder or non-responder cells (Figures 3K and 3L). Taken together, our results indicate that, in addition to re-epithelialization, Piezo also regulates the inflammatory response by maintaining homeostatic calcium levels across the epidermis and thereby ensures sufficiently high levels of damage-induced ROS.

### Loss of Piezo compromises epithelial barrier function and reduces post-wounding survival

**Figure 4:**
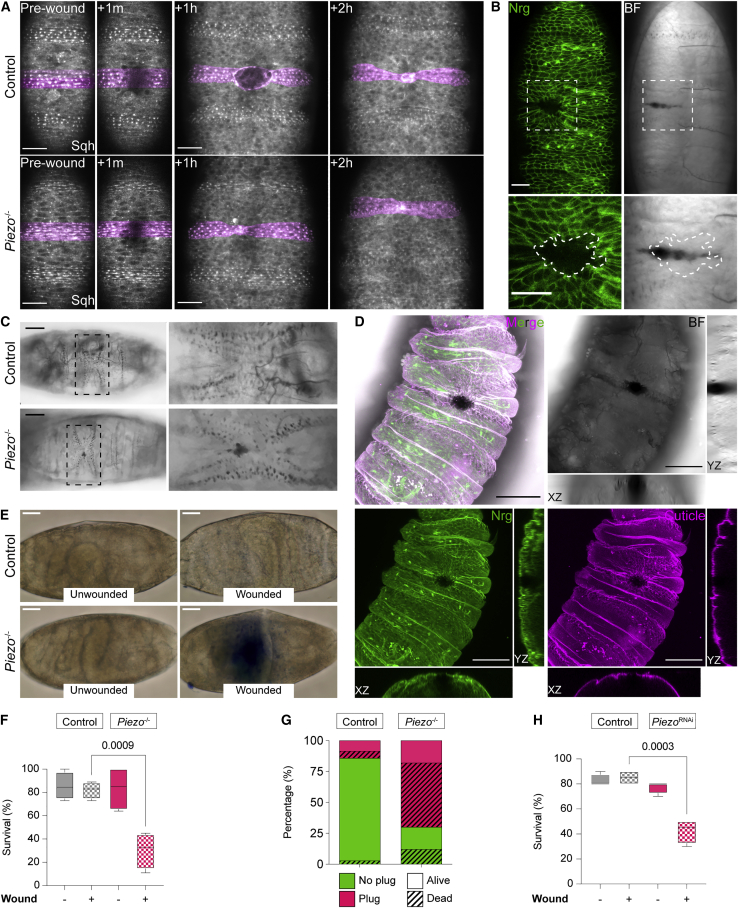
Loss of Piezo compromises epithelial barrier function after wounding (A) Confocal images of embryos expressing Sqh-GFP (gray) to label a ventral denticle belt (pseudo-colored in magenta), severed by wounding (+1 min). Recovery of tissue architecture is shown at 1 h and 2 h post-wounding. (B) *Piezo*^-/-^ embryo-to-L1 larva imaged ∼7 h post-wounding. A gap in the epidermis (Nrg, green) is highlighted by the dashed square; a melanotic plug is shown in brightfield. Dashed magenta line: epithelial gap outline. (C) Embryo-to-L1 larvae imaged by brightfield to highlight the original site of wounding (black rectangles), with a persisting melanotic plug observed in *Piezo*^-/-^ embryos. (D) *Piezo*^-/-^ L1 larva imaged ∼24 h post-wounding. A large melanotic plug (brightfield) is present at the wound site, accompanied by a compromised underlying epithelium (Nrg, green) and defective cuticle (autofluorescence, magenta). (E) Permeability of control and *Piezo*^-/-^ embryos prior to and upon wounding. (F) Survival rate of unwounded and wounded control and *Piezo*^-/-^ embryos. (G) Correlation analysis of melanotic plug persistency and lethality. (H) Survival rate of unwounded and wounded control and *Piezo*^RNAi^ embryos. Scale bars, (A) (B): 20 μm; (C) (D) (E): 50 μm. Error bars: SEM. Related to Figure S4.

The negative regulation of Piezo on wound closure seems counterintuitive: why would evolution favor the expression of a molecule that delays damage repair? One explanation might be that slower closure is necessary to maintain epithelial patterning during wound repair. To test this hypothesis, we made wounds that severed a single denticle belt (a myosin-rich pattern present on the ventral embryonic epidermis) and assessed the ability of *Piezo*^-/-^ embryos to reconstitute it during re-epithelialization. Denticle alignment post-repair was indistinguishable between control and *Piezo*^-/-^ embryos (Figure 4A), indicating that loss of Piezo does not compromise the ability of closing wounds to maintain tissue geometry. Another plausible explanation for the requirement of a molecular brake during wound healing is that, despite being initially beneficial, a rapid re-epithelialization may be detrimental in the long-term. To explore this possibility, we performed long-term live imaging to study the evolution of wounded tissues in control and *Piezo*^-/-^ embryos as they proceeded through development. This revealed that, beginning at 4–6 hours post-wounding, damaged *Piezo*^-/-^ epithelia degenerate into a large epidermal gap (Figures 4B and S4A)—a phenomenon rarely seen in control animals. Furthermore, while control embryos tend to develop small and rapidly resolving melanotic plugs—scab-like structures typically observed during the late larval damage response^13^—epithelial gaps observed in *Piezo*^-/-^ embryos failed to resolve and became accompanied by a persistent and progressively large melanotic plug at the original wound site (Figures 4B, 4C, and S4A). Accordingly, while wounded control embryos develop into larvae that are indistinguishable from unwounded animals, a large proportion of wounded *Piezo*^-/-^ embryos develop into larvae exhibiting epithelial discontinuity, a prominent melanotic plug, and a failure to assemble the cuticle layer at the original wound site (Figure 4D). This epidermal breach represents a clear compromise in epithelial barrier function, as evidenced by the dramatic increase in leakiness of a cell-impermeable dye in wounded *Piezo*^-/-^ embryos (Figure 4E). Importantly, we found no difference in the permeability of unwounded embryos, suggesting that loss of Piezo per se does not compromise steady-state epithelial barrier function. To understand the repercussions of this impaired barrier function on development and overall animal wellbeing, we measured post-wounding survival, which showed that a strikingly higher proportion of wounded *Piezo*^-/-^ embryos die during the earlier stages of development in comparison to wounded controls (Figure 4F). Importantly, in agreement with the reported non-essential role of Piezo during development,^14^ we found no difference in the survival rate of unwounded embryos. To understand whether the higher mortality of wounded *Piezo*^-/-^ embryos is directly linked to the impaired epithelial barrier function, we carried out a correlation analysis between persistence of a melanotic plug—macroscopic readout of defective epidermal integrity—and lethality. As shown in Figure 4G, we found that formation of a melanotic plug is a reliable predictor of lethality. Lastly, we sought to understand whether depleting *Piezo* expression specifically within the epithelium (*Piezo*^RNAi^) was sufficient to affect post-wounding survival. As shown in Figure 4H, we found that a significantly larger proportion of wounded *Piezo*^RNAi^ embryos failed to reach adulthood in comparison to wounded control embryos. Again, epidermal Piezo depletion did not affect survival of unwounded embryos. Altogether, our data show that, despite closing at a faster rate, wounds inflicted on *Piezo*^-/-^ embryos fail to fully resolve and restore epidermal integrity. The resulting disrupted barrier function hinders normal development post-wounding, frequently resulting in lethality. Given that development entails morphogenetic movements that exert a strain on the outermost tissues, we speculate that fast-sealing wounds do not confer the ability to withstand repetitive mechanical strain, therefore compromising epithelial resilience. The epidermis represents the interface between an organism and the outside world. Due to its intrinsically exposed location, this tissue is constantly subjected to injuries throughout every organism’s life; being able to repair them is an evolutionarily widespread prerogative. It is only natural, given their life-threatening potential, to assume that wounds must be closed within the shortest time-frame possible. Our work, however, clearly demonstrates that—*in vivo*—wounds are dealt with using a “slow and steady wins the race” approach.

## STAR★Methods

### Key resources table

**Table:** 

REAGENT or RESOURCE	SOURCE	IDENTIFIER
**Antibodies**		

α-GFP	Abcam	Cat# ab13970; RRID: AB_300798
α-chicken AF488	Invitrogen	Cat# A-11039; RRID: AB_142924

**Chemicals, peptides, and recombinant proteins**		

Voltalef oil	VWR	Cat# 24627.188
Vectashield	Vector labs	Cat# H-1000 RRID: AB_2336789
Heptane	Sigma	Cat# 246654
Formaldehyde 16%	MP Bio	Cat# 199983
Methanol	Sigma	Cat# 34860
DMSO	Merck Life Sciences	Cat# 20-139
Heptane	Sigma	Cat# 34873
Amplex™ UltraRed Reagent	Invitrogen	Cat# A36006
Methylene Blue	Sigma	Cat# MB-1
Diethyl Ether	Fisher scientific	Cat# 10696442
Femtotips	Eppendorf	Cat# 930000035

**Experimental models: Organisms/strains**		

*D. melanogaster*: Piezo^-/-^:w[^∗^]; PBac{w[+mC]=RB5.WH5}Piezo[KO]	Bloomington Drosophila Stock Center	RRID: BDSC_58770; FlyBase: FBti0147345
*D. melanogaster*: Nrg:w[1118] P{w[+mC]=PTT-GA}Nrg[G00305]	Bloomington Drosophila Stock Center	RRID: BDSC_6844; FlyBase: FBti002785
*D. melanogaster*: 69B-GL4:w[^∗^]; P{w[+mW.hs]=GawB}69B	Bloomington Drosophila Stock Center	RRID: BDSC_1774; FlyBase: FBti0002093
*D. melanogaster*: LexA^RNAi^:y[1] sc[^∗^] v[1] sev[21]; P{y[+t7.7] v[+t1.8]=TRiP.HMS05768}attP40	Bloomington Drosophila Stock Center	RRID: BDSC_67942; FlyBase: FBti018675
*D. melanogaster*: LexA^RNAi^:y[1] v[1]; P{y[+t7.7] v[+t1.8]=TRiP.HMS05775}attP2	Bloomington Drosophila Stock Center	RRID: BDSC_67946; FlyBase: FBti0186764
*D. melanogaster*: Piezo^RNAi^:P{KK111660}VIE-260B	Vienna Drosophila Resource Center	RRID: FlyBase_FBst0474309
*D. melanogaster*: Piezo^MiMIC^:y[1] w[67c23]; Mi{PT-GFSTF.0}Piezo[MI04189-GFSTF.0]	Bloomington Drosophila Stock Center	RRID: BDSC_60209; FlyBase: FBti0178581
*D. melanogaster*: PMCA^RNAi^:y[1] v[1]; P{y[+t7.7] v[+t1.8]=TRiP.JF01145}attP2	Bloomington Drosophila Stock Center	RRID: BDSC_31572; FlyBase: FBti0130608
*D. melanogaster*: UAS-R-Geco:w[^∗^]; PBac{y[+mDint2] w[+mC]=20XUAS-IVS-NES-jRGECO1a-p10}VK00005/TM6B, Tb[1]	Bloomington Drosophila Stock Center	RRID: BDSC_63794; FlyBase: FBti0180190
*D. melanogaster*: Srp-MoeCherry:*w[1118]; P{w[+mC]=srpHemo-Moe.3XmCherry}3*	Bloomington Drosophila Stock Center	RRID: BDSC_78362; FlyBase: FBti0197718
*D. melanogaster*: Sqh-GFP:w[1118]; P{w[+mC]=sqh-GFP.RLC}3	Bloomington Drosophila Stock Center	RRID: BDSC_57145; FlyBase:FBti0150058

**Software and algorithms**		

GraphPad Prism V9.2.0	GraphPad Software	https://www.graphpad.com/scientific-software/prism/
ImageJ/Fiji V2.3.0/1.53f	National Institute of Health	https://imagej.nih.gov/ij/
Zen Black	Zeiss	https://www.zeiss.com/microscopy/int/products/microscope-software/zen.html
Illustrator	Adobe	https://www.adobe.com/uk/products/illustrator.html
Photoshop	Adobe	https://www.adobe.com/uk/products/photoshop.html

### Resource availability

#### Lead contact

Further information and requests for resources and reagents should be directed to and will be fulfilled by the lead contact, William Wood (w.wood@ed.ac.uk).

#### Materials availability

• This study did not generate new unique reagents.
• Fly strains used in this study are available from the lead contact upon request.

### Experimental model and subject details

#### Drosophila strains and husbandry

All *Drosophila* strains were raised at 25°C on standard cornmeal-agar food at 50%–60% relative humidity in a 12:12 h light:dark cycle. All genotypes used in this study are listed in Methods S1B.

### Method details

#### *Drosophila* embryo injection and wounding

Embryos were collected from apple juice agar plates from overnight laying cages maintained at 25° C. Embryos were collected in cell strainers, dechorionated in bleach for 90 s and washed repeatedly with distilled water. Stage 15 embryos (stage 14 to visualize dorsal closure) were manually selected and mounted ventral side up on a glass slide with double-sided sticky tape (dorsal side up to visualize dorsal closure and mounted on glass bottom dishes to image late embryo/L1 larvae), embedded in VOLTALEF oil^15^, and covered with No 1.0 coverslip (SLS). For injection, embryos were dehydrated for 15 minutes on silica beads. Injection was performed using Femtotip II (Eppendorf) on a Femtojet Injectman Rig (Eppendorf). Epithelial wounds were generated using laser ablation (nitrogen-pumped micropoint ablation laser tuned to 435 nm, Andor Technologies) as previously described^2^.

#### Imaging, image processing, and analysis

Confocal imaging was performed on a Zeiss LSM880 laser scanning confocal microscope equipped with a 40x/1.3 oil immersion objective. GFP and mCherry were excited at 488 and 561 nm respectively; the cuticle autofluorescence was obtained by exciting at 405 nm. Images were imported into Fiji^16^ and processed as required. To aid late embryo/L1 live imaging, animals were sedated by exposure to Diethyl Ether vapors.

#### Wound closure

To monitor wound closure, 11 μm deep Z-stacks were acquired every 30 seconds, and the wound area measured at regular intervals (5 frames= 2.5 minutes) using the Fiji Freehand selections tool. Wound area values were then exported to excel to calculate T_50%_ and rate of wound closure. T_50%_ values were calculated performing a polynomial interpolation on wound area values (R^2^_Control_= 0.986 ± 0.008, R^2^_*Piezo*-/-_= 0.982 ± 0.016, Mean ± S.D.) Rate of wound closure indicate the μm^2^ of wound area that are lost per minute as the wound closes and reaches 75% of closure.

#### Epithelial cell number

Embryonic epithelial cell number was calculated by manually counting the number of cells within three equally-sized squares placed on comparable positions across different embryos.

#### Epithelial cells intercalation

Individual epithelial cells were tracked using the MTrackJ Fiji plugin^17^ from t= 5 minutes post-wound (earliest time at which the wound edge is clearly identifiable) to t= 90 minutes. Intercalating cells were defined as those that lose contact with the initially neighboring cells. Intercalation time corresponds to the time (in minutes) required for individual epithelial cells to fully move past neighboring cells.

#### Wound ellipticity

Wound ellipticity at given time-points (5-, 15-, 30-, 45-, 60- and 75-minutes post-wounding), was defined by the ratio between the wound area and the area of the smallest ellipse (defined by the Fiji Elliptical selection tool) able to contain the wound. Consequently, an ellipticity of 1 indicates a perfectly elliptic wound, lower values indicate a progressively more irregular shape.

#### Myosin wound-edge measurements

Absolute wound edge myosin fluorescence intensity values were obtained using the Fiji freehand Line tool. To measure wound edge myosin heterogeneity, each wound was divided into 8 equally-long segments (1-8), and the averaged myosin intensity along each segment was normalized to the averaged wound edge myosin intensity; normalized values were then ranked (lowest to highest) and plotted as heatmaps. To obtain the myosin heterogeneity score, we compared the SD of the wound edge myosin distribution across the 8 segments. To visually represent wound edge myosin heterogeneity, myosin intensity was plotted along a set length of wound edge, and the lowest and the highest myosin intensity values then connected by differentially-colored rectangles; progressively taller rectangles correspond to a more widely-spread myosin intensity values.

#### Tissue tension measurement

Cortical myosin was used as epithelial tension readout. Average cortical myosin fluorescence intensity values were obtained using the Fiji freehand Line by outlining ten non-neighboring epithelial cells within individual embryos.

#### Calcium signal

To measure R-Geco calcium signal, 14 μm deep Z-stacks were acquired every 5 seconds. Regions of interest (ROIs) were manually selected on average intensity projections, and average pixel intensity values (basal and peak) of the ROI obtained using the time series V3.0 Fiji plugin. To measure GCaMP3 calcium signal, 4 μm deep Z-stacks were acquired every 5 seconds, and ROIs manually selected on average intensity projections. Basal calcium intensity was obtained from the pre-wound frame, peak calcium intensity was obtained from the brightest time-frame. Peak ΔF/F_0_ was calculated as difference of the average peak fluorescence intensity and the basal intensity (F_peak_-F_basal_), divided by the basal intensity (F_basal_) values.

#### Damage-induced ROS production

To monitor ROS levels upon wounding, 5 μm deep Z-stacks were acquired every 30 seconds and ROIs manually selected on average intensity projections. For each time-point, the ROI absolute fluorescence intensity (ROI_Absolute_) was calculated (ROI_Mean_^∗^ ROI Area) and normalized against the corrected background fluorescence intensity [Background_Corrected_= (Background_Mean_^∗^ Background Area) – ROI_Absolute_].

#### Macrophage recruitment to wound

Individual macrophages were tracked throughout closure using the MTrackJ Fiji plugin, and resulting tracks analyzed using the Chemotaxis tool (ibidi GmbH) Fiji plugin to obtain individual macrophages speed values.

#### Embryo permeability test

Stage 15 embryos were dechorionated as described above and then devitellinized in Heptane for 15 minutes. Embryos were mounted ventral side up on a glass slide in oil, wounded as described above, kept at 25° for 4 hours, collected from the wounding slides and then submerged in a Methylene Blue solution (25 mM in distilled water) for 1 hour. Embryos were then extensively washed in distilled water and imaged on a EVOS XL Core microscope equipped with 40X objective.

#### Embryo survival after wound and larval imaging

Stage 15 embryos mounted and subjected to either wound or mock wound were allowed to recover for 1 h at 18°C. Embryos were then detached from the microscope slide, placed on apple plates, kept at 25° and monitored for the following 10 days to record the adult emergence rate. Alternatively, collected embryos were individually placed on apple plates to monitor their development in the following 24 hours. L1 larvae were snap frozen in dry ice before microscopic observation.

#### Drosophila embryo fixation and immunostaining

Dechorionated embryos were fixed in 1:1 4% PFA:heptane mixture for 30 minutes at room temperature, washed with PBS-Tx-BSA and incubated in primary antibodies at 4°C overnight. Embryos were incubated with secondary antibodies for 1 hour at room temperature. Washed embryos were then mounted in Vectashield mounting medium. Primary antibodies: α-GFP (1:500, Abcam Ab13970), Secondary antibodies: α-chicken AF488 (1:200 Invitrogen A11039).

### Quantification and statistical analysis

#### Statistical analysis

All datasets underwent Shapiro-Wilk normality tests to ensure that the appropriate statistical tests were performed. Two-tailed unpaired t-tests and Mann-Whitney tests were then performed on normally-distributed and non-normally-distributed data respectively. Datasets with more than two groups were compared using ANOVA tests; for data with comparable variances (F-tested) Tukey’s or Sidak’s multiple comparisons were performed, as recommended by the GraphPad Prism V8.4.1 software. All graphs show mean ± SEM. Statistical details can be found in Method S1A.

## Data Availability

• All data reported in this paper will be shared by the lead contact upon request. • This study did not generate original code. • Any additional information required to reanalyze the data reported in this paper is available from the lead contact upon request.

## Supplemental information

Document S1. Figures S1–S4

Document S2. Methods S1A and S1B Methods S1A. Statistical details of the experiments. Number of samples (N) and statistical tests performed for each experiment described in this study. N refers to the number of embryos, epithelial cells or macrophages, as indicated. Methods S1B: Genotypes list. Complete list of the Drosophila genotypes used in this study

Document S3. Article plus supplemental information
