# Supplementary material for: Piezo acts as a molecular brake on wound closure to ensure effective inflammation and maintenance of epithelial integrity
Source: Curr Biol. 2022 Aug 22;32(16):3584–3592.e4. doi: 10.1016/j.cub.2022.06.041 (PMC9616804; doi:10.1016/j.cub.2022.06.041)
Supplement: Document S1. Figures S1–S4 [file mmc1.pdf]

Current Biology, Volume *32*

Supplemental Information

Piezo acts as a molecular brake on wound closure to ensure effective inflammation and maintenance of epithelial integrity

Luigi Zechini, Clelia Amato, Alessandro Scopelliti, and Will Wood

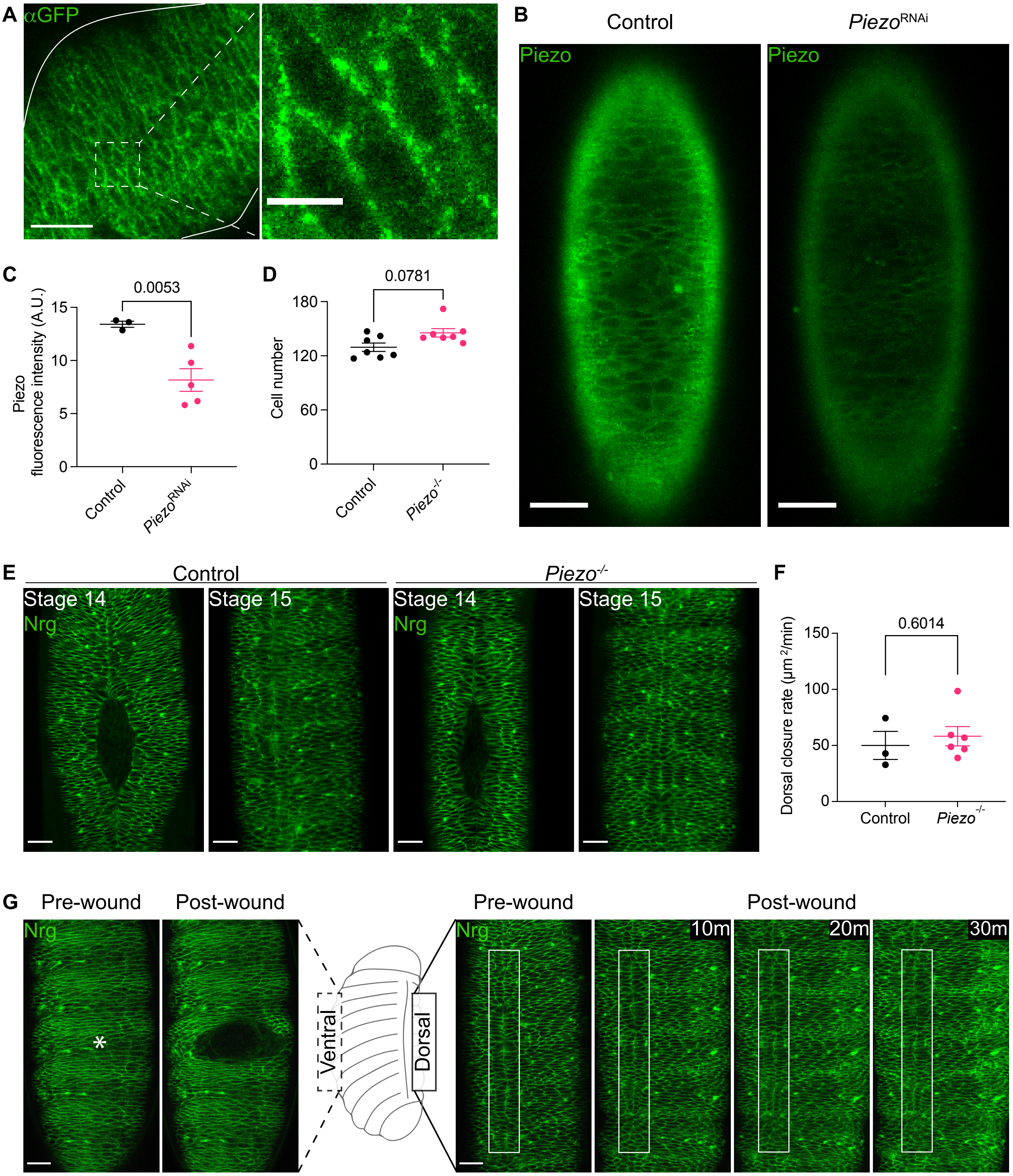

Figure S1. Piezo epidermal expression, *Piezo* RNAi effectiveness, epidermal cell density and dorsal closure. Related to figure 1. A. Representative immunofluorescent staining of stage 15 Piezo^{MiMIC} embryo. Scale bar: 20μm, inset scale bar: 10 μm. **B.** Representative confocal images of stage 15 Piezo^{MiMIC} embryo showing the RNAi efficiency. Scale bar: 20μm. **C.** Quantification of the fluorescence intensity as in B. **D.** Quantification of epidermal cell density in control and *Piezo*$^{-/-}$ stage 15 embryos. **E.** Representative confocal images of the dorsal epidermis of stage 14 and 15 control and *Piezo*$^{-/-}$ embryos showing the completion of dorsal closure. Scale bar: 20μm. **F.** Quantification of dorsal closure rate in control and *Piezo*$^{-/-}$ embryos. **G.** Confocal images of ventral and dorsal epithelia of the same *Piezo*$^{-/-}$ embryo, showing that no changes to sealed dorsal hole (white rectangles) occurs following wounding on the ventral epidermis (asterisk: wound). Scale bar: 20μm.

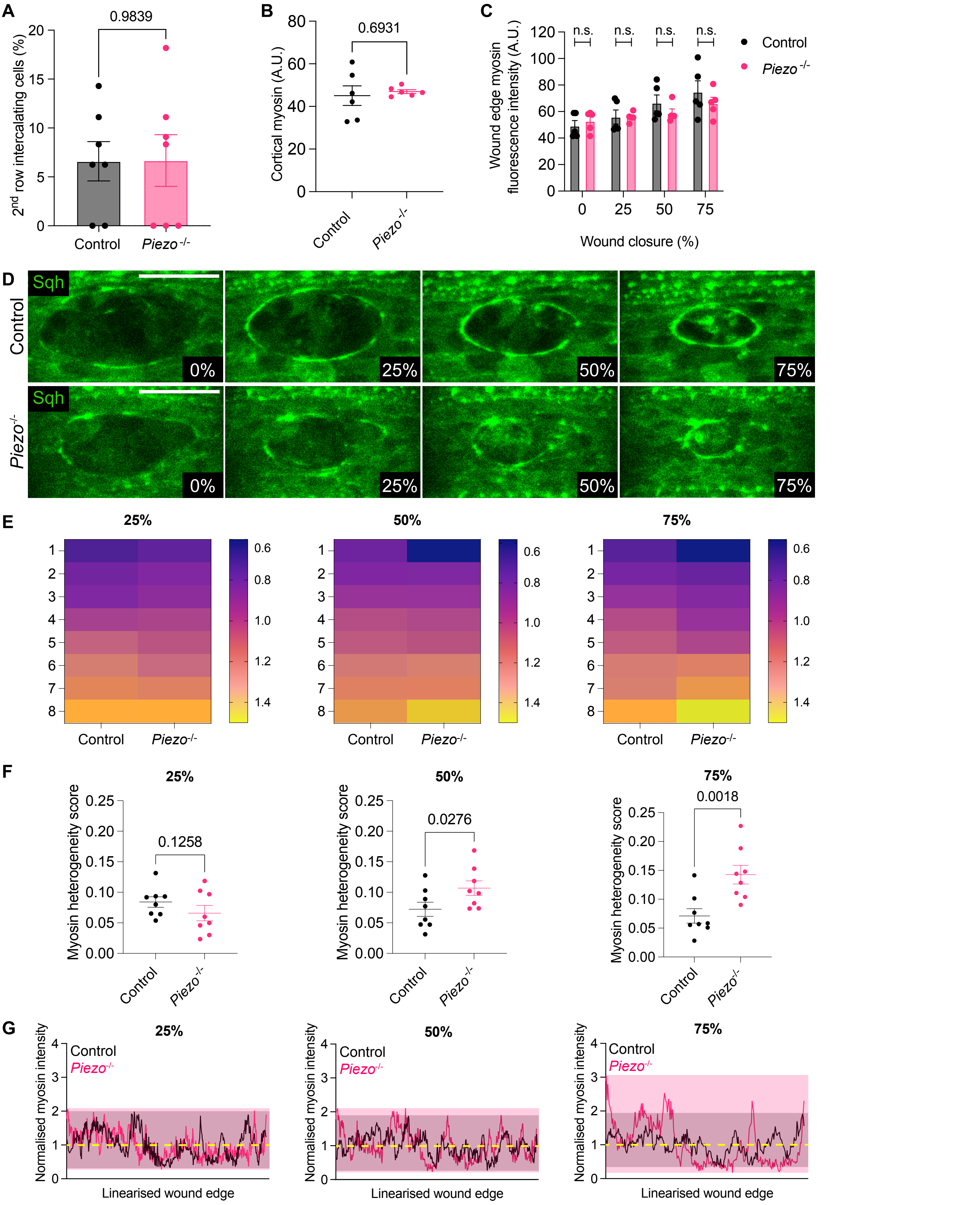

Figure S2. Loss of Piezo increases localised tissue fluidity and exacerbates wound edge myosin heterogeneity. Related to figure 2. A. Quantification of intercalations two rows from the wound edge. **B.** Quantification of cortical myosin fluorescence intensity in unchallenged epidermises. **C.** Quantification of wound edge myosin fluorescence intensity at different percentages of closure. **D.** Representative images of wound edge myosin fluorescence intensity at different percentages of closure. Scale bar: 20μm. **E.** Heatmaps of wound edge myosin heterogeneity at different percentages of closure. **F.** Myosin heterogeneity score at different percentages of wound closure. **G.** Representative fluorescence plots showing myosin heterogeneity at different percentages of wound closure. Rectangles connect the lowest to the highest values. Control: black; *Piezo*$^{-/-}$: magenta.

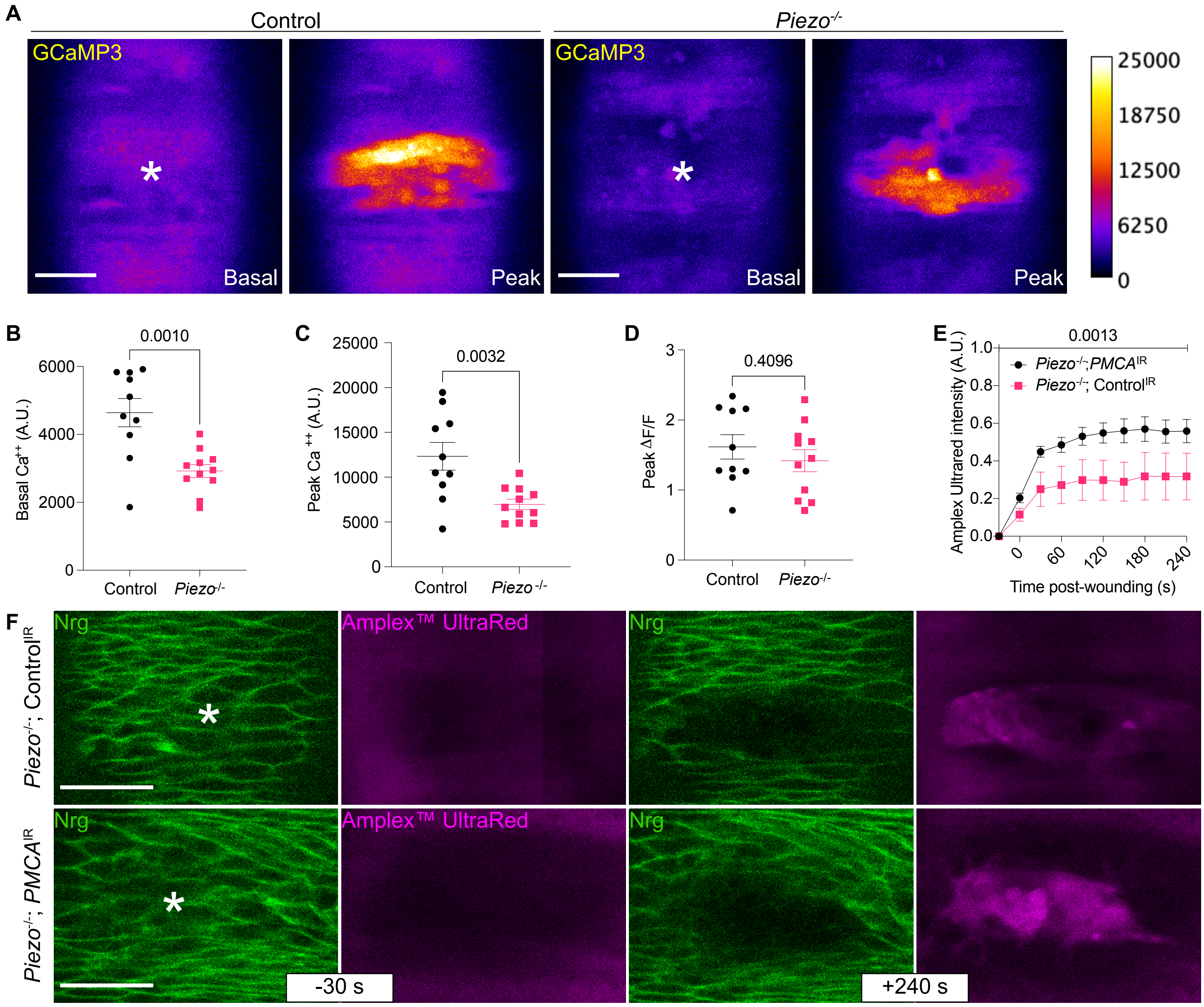

Figure S3. Effects of loss of Piezo on calcium and damage-induced ROS production. Related to figure 3. A. Confocal images of basal (pre-wounding) and peak (post-wounding) calcium flux across the ventral epithelium of stage 15 embryos expressing the GCamP3 calcium reporter. Asterisks: wound. Scale bar: 20µm. **B.** Quantification of basal (pre-wounding) calcium levels. **C.** Quantification of peak (post-wounding) calcium levels. **D.** Quantification of normalized peak (post-wounding) calcium levels. **E.** Normalised Amplex™ UltraRed intensity fluorescence prior to- and post-wounding. **F.** Confocal images of unwounded (-30 s) and wounded (+240 s) *Piezo*$^{-/-}$; *PMCA*^{IR} and *Piezo*$^{-/-}$; Control^{IR} embryos expressing an epithelial marker (Nrg, green) and injected with Amplex™ UltraRed (magenta); asterisk: wound.

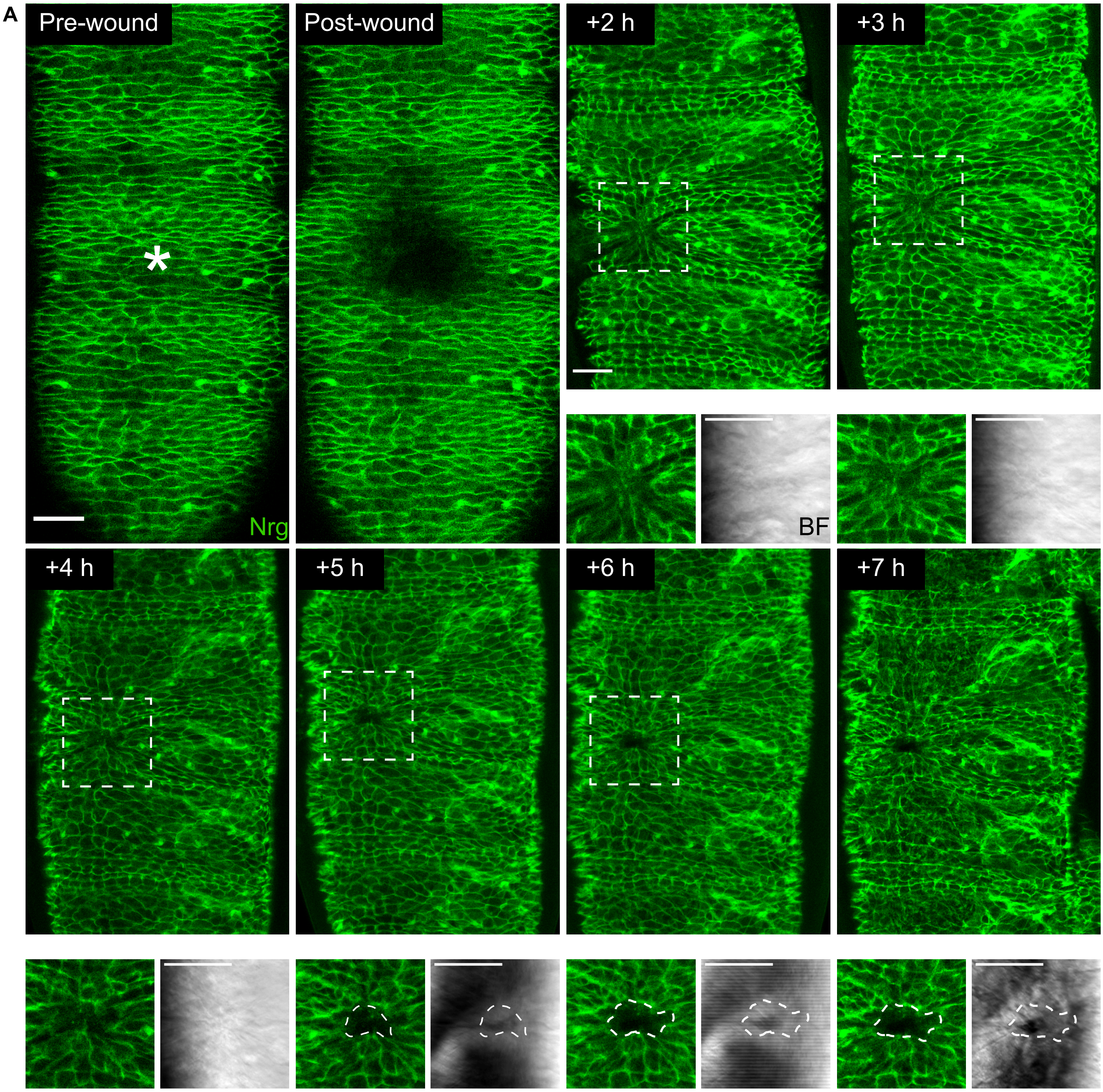

Figure S4. Wound healing progression upon loss of Piezo. Related to figure 4. A. *Piezo*$^{-/-}$ embryo-to-L1 larva imaged prior to wounding (Pre-wound), immediately after wounding (Post-wound), and at regular 1 hour intervals up to 7 hours post-wounding. The damaged area is indicated by the dashed square and shown in greater details within the insets. A gap in the epidermis (Nrg, green) and a melanotic plug (BF) are highlighted by the dashed white outline. Asterisks: wound. Scale bar: 20µm.
