# Supplementary material for: Piezo acts as a molecular brake on wound closure to ensure effective inflammation and maintenance of epithelial integrity
Source: Curr Biol. 2022 Aug 22;32(16):3584–3592.e4. doi: 10.1016/j.cub.2022.06.041 (PMC9616804; doi:10.1016/j.cub.2022.06.041)
Supplement: Document S2. Methods S1A and S1B — Methods S1A. Statistical details of the experiments. Number of samples (N) and statistical tests performed for each experiment described in this study. N refers to the number of embryos, epithelial cells or macrophages, as indicated. Methods S1B: Genotypes list. Complete list of the Drosophila genotypes used in this study [file mmc2.pdf]

Methods S1A: Statistical Analysis		
Figure	N	Statistical analysis
1B, D	Control: 7<br>*Piezo*$^{-/-}$: 7	Unpaired t-test
1C	Control: 7<br>*Piezo*$^{-/-}$: 7	Kolmogorov-Smirnov test
1E	Control: 8<br>*Piezo*$^{-/-}$: 10	Unpaired t-test
1G, I, J	Control: 5<br>*Piezo*^{RNAi}: 5	Unpaired t-test
1H	Control: 7<br>*Piezo*^{RNAi}: 7	Kolmogorov-Smirnov test
2B	Control: 10<br>*Piezo*$^{-/-}$: 10	Mann-Whitney test
2C	Control: 10 (11 intercalation events)<br>*Piezo*$^{-/-}$: 10 (36 intercalation events)	Unpaired t-test
2E	Control: 10 (11 intercalation events)<br>*Piezo*$^{-/-}$: 10 (36 intercalation events)	2way ANOVA (A-P vs D-V)
2F	Control: 10<br>*Piezo*$^{-/-}$: 10	2way ANOVA (Control vs *Piezo*$^{-/-}$ at given time-points).
2H	Control: 5<br>*Piezo*$^{-/-}$: 5	n/a
3B-D	Control: 9<br>*Piezo*$^{-/-}$: 10	Unpaired t-test
3E	Control: 4<br>*Piezo*$^{-/-}$: 4	Kolmogorov-Smirnov test
3I-K	Control: 10<br>*Piezo*$^{-/-}$: 10	Unpaired t-test
3L	$\text{Control}_{\text{Responders}}$: 93<br>$\text{Control}_{\text{Non-Responders}}$: 112<br>*Piezo*$^{-/-}_{\text{Responders}}$: 71<br>*Piezo*$^{-/-}_{\text{Non-Responders}}$: 140	Mann-Whitney test
4F	$\text{Control}_{\text{Unwounded}}$: 50<br>$\text{Control}_{\text{Wounded}}$: 48<br>*Piezo*$^{-/-}_{\text{Unwounded}}$: 51<br>*Piezo*$^{-/-}_{\text{Wounded}}$: 50	Unpaired t-test
4G	$\text{Control}_{\text{Alive/No Plug}}$: 29<br>$\text{Control}_{\text{Alive/Plug}}$: 3<br>$\text{Control}_{\text{Dead/No Plug}}$: 1<br>$\text{Control}_{\text{Dead/ Plug}}$: 2<br>*Piezo*$^{-/-}_{\text{Alive/No Plug}}$: 9<br>*Piezo*$^{-/-}_{\text{Alive/Plug}}$: 9<br>*Piezo*$^{-/-}_{\text{Dead/No Plug}}$: 6<br>*Piezo*$^{-/-}_{\text{Dead/ Plug}}$: 26	n/a
4H	$\text{Control}_{\text{Unwounded}}$: 40<br>$\text{Control}_{\text{Wounded}}$: 40<br>*Piezo*$^{RNAi}_{\text{Unwounded}}$: 40<br>*Piezo*$^{RNAi}_{\text{Wounded}}$: 40	Unpaired t-test
S1C	Control: 3<br>*Piezo*^{RNAi}: 5	Unpaired t-test

S1D	Control: 7<br>*Piezo*^{RNAi}: 7	Mann-Whitney test
S2A	Control: 7<br>*Piezo*^{RNAi}: 7	Unpaired t-test
S2B	Control: 6<br>*Piezo*^{RNAi}: 6	Unpaired t-test
S2C	Control: 5<br>*Piezo*^{RNAi}: 5	Mann-Whitney test/<br>Unpaired t-test
S2F	Control: 5<br>*Piezo*^{RNAi}: 5	Unpaired t-test
S3B-D	Control: 10<br>*Piezo*$^{-/-}$: 11	Unpaired t-test
S3E	PMCA^{IR}: 6<br>Control^{IR}: 5	Kolmogorov-Smirnov test

Methods S1B: Genotypes		
**Figure**	**Panel**	**Genotypes**
Figure 1	A-E	Nrg; +; srp-MoeCherry<br>Nrg; Piezo^{KO}; srp-MoeCherry
	F-J	Nrg; LexA IR; 69B-gal4<br>Nrg; Piezo IR; 69B-gal4
Figure 2	A-E	Nrg; +; srp-MoeCherry<br>Nrg; Piezo^{KO}; srp-MoeCherry
	F-H	+; Sqh-GFP<br>Piezo^{KO}; Sqh-GFP
Figure 3	A-D	Nrg; +; 69B-GAL4, UAS-R-Geco<br>Nrg; Piezo^{KO}; 69B-GAL4, UAS-R-Geco
	E-F	+; Sqh-GFP<br>Piezo^{KO}; Sqh-GFP
	G-L	Nrg; +; srp-MoeCherry<br>Nrg; Piezo^{KO}; srp-MoeCherry
Figure 4	A	+; Sqh-GFP<br>Piezo^{KO}; Sqh-GFP
	B	Nrg; Piezo^{KO}; srp-MoeCherry
	C-G	Nrg; +; srp-MoeCherry<br>Nrg; Piezo^{KO}; srp-MoeCherry
	H	Nrg; LexA IR; 69B-GAL4<br>Nrg; Piezo IR; 69B-GAL4
Figure S1	A	Piezo^{MiMIC}
	B-C	LexA IR/ Piezo^{MiMIC}; 69B-GAL4/+<br>Piezo IR/ Piezo^{MiMIC}; 69B-GAL4/+
	D-G	Nrg; +; srp-MoeCherry<br>Nrg; Piezo^{KO}; srp-MoeCherry
Figure S2	A	Nrg; +; srp-MoeCherry<br>Nrg; Piezo^{KO}; srp-MoeCherry
	B-G	+; Sqh-GFP<br>Piezo^{KO}; Sqh-GFP
Figure S3	A-D	+; 69B-GAL4, UAS-GCamp3<br>Piezo^{KO}; 69B-GAL4, UAS-GCamp3
	E-F	Nrg; Piezo^{KO}; 69B-GAL4/LexA IR<br>Nrg; Piezo^{KO}; 69B-GAL4/PMCA IR
Figure S4	A	Nrg; Piezo^{KO}; srp-MoeCherry
Movie S1		Nrg; +; srp-MoeCherry<br>Nrg; Piezo^{KO}; srp-MoeCherry
Movie S2		Nrg; LexA IR; 69B-gal4<br>Nrg; Piezo IR; 69B-gal4
Movie S3		Nrg; +; 69B-GAL4, UAS-R-Geco<br>Nrg; Piezo^{KO}; 69B-GAL4, UAS-R-Geco
Movie S4		Nrg; +; srp-MoeCherry<br>Nrg; Piezo^{KO}; srp-MoeCherry
